# The Roles of School Readiness and Poverty-Related Risk for 6^th^ Grade Outcomes

**DOI:** 10.5539/jedp.v6n1p140

**Published:** 2016-02-22

**Authors:** Emily Pressler, C. Cybele Raver, Allison H. Friedman-Krauss, Amanda Roy

**Affiliations:** 1 Department of Applied Psychology, Steinhardt School of Culture, Education, and Human Development, New York University, New York, USA; 2 National Institute for Early Education Research, Rutgers University, New Brunswick, New Jersey, USA; 3 Department of Psychology, University of Illinois at Chicago, Chicago, Illinois, USA

**Keywords:** cumulative risk, retention, school readiness, suspension

## Abstract

Low-income students are at increased risk for grade retention and suspension, which dampens their chances of high school graduation, college attendance, and future success. Drawing from a sample of 357 children and their families who participated in the Chicago School Readiness Project, we examine whether greater exposure to cumulative poverty-related risk from preschool through 5^th^ grade is associated with greater risk of student retention and suspension in 6^th^ grade. Logistic regression results indicate that exposure to higher levels of cumulative risk across the elementary school years is associated with students’ increased risk of retention in 6^th^ grade, even after controlling for child school readiness skills and other covariates. Importantly, findings of the association between average cumulative risk exposure and student suspension are more complex; the role of poverty-related risk is reduced to non-significance once early indicators of child school readiness and other covariates are included in regression models. While, children’s early externalizing behavior prior to kindergarten places children at greater risk of suspension 7 years later, children’s higher levels of internalizing behaviors and early math skills are associated with significantly decreased risk of suspension in the 6^th^ grade. Together, findings from the study suggest the complex ways that both early school readiness and subsequent exposure to poverty-related risk may both serve as compelling predictors of children’s likelihood of “staying on track” academically in the 6^th^ grade.

## 1. Introduction

Much research finds lower income students lag behind their higher income peers in cognitive and social-emotional skills by school entry. These initial poverty-related gaps in student skills increase as children age and may have increased over the last several decades (e.g., Duncan & Magnuson, 2011; Reardon, 2011). In addition, poverty-related disparities in student functioning extend beyond achievement to rates of retention and out-of-school suspension, which are more common events for children from lower income households relative to their higher income peers (Child Trends, 2012; U.S. Department of Education Office for Civil Rights, 2014).

One strategy for closing these gaps has been to invest in children’s early educational experiences through provision of early childhood educational programs such as universal pre-K and Head Start. In national impact evaluations, programs such as Head Start have been found to improve children’s school readiness (U.S. Department of Health and Human Services, 2010). However, in Head Start’s national impact evaluation, as well as in many other efficacy trials (including our own Chicago School Readiness Project), early gains in child outcomes are hard to maintain over time, suggesting that the benefits of early educational programming “fade out”. Rather than viewing these fade out findings as evidence that preschool interventions do not work, an alternative and plausible perspective may be that low-income children often face stressors inside and outside of school that make it less likely that any early educational gains “stick” over time.

The current study’s overarching aim is to integrate two common approaches to understanding income-based gaps in student educational outcomes. The first approach, rooted in developmental science, focuses on children’s behavioral and academic trajectories as key predictors of their likelihood for retention and suspension while simultaneously placing less emphasis on the environmental risks that may alter those trajectories (e.g., Reinke, Herman, Petras, & Ialongo, 2008). The second approach focuses on the environmental contexts, such as poverty, that support or constrain children’s academic success and places less emphasis on individual differences in children’s behavioral or academic profiles and the role that children play in shaping their experiences within school (e.g., Duncan, Morris, & Rodrigues, 2011; Duncan, Yeung, Brooks-Gunn, & Smith, 1998). To achieve our overarching goal, we include characteristics of children (i.e., academic and behavioral school readiness prior to kindergarten entry) and their environments (i.e., average cumulative risk from kindergarten through elementary school) to obtain clearer estimates of the contributions each has to child school success in 6^th^ grade. In the following sections, we first briefly review recent findings on the significance of retention and suspension for children’s long-term success. We then turn to models of poverty and poverty-related risk to place the events that may lead children to retention and suspension in middle childhood within a broader socioeconomic context. Last, we consider the role children’s earlier academic and behavioral skills play in their risk for retention and suspension in the middle grades.

### 1.1 Retention and Suspension in the Middle Grades

Grade retention and suspension are culminating events in a student’s life that can alter the course of their later academic success. Therefore Chicago Public Schools (CPS) promotion and retention policies draw from multiple sources of information, specifically student grades, test scores, and attendance records to determine which students should be promoted or retained. Not surprisingly, these same indicators are also linked to longer-term educational and occupational outcomes. For example, The Consortium on Chicago School Research finds that student GPA and attendance in the 6^th^, 8^th^, and 9^th^ grades are predictive of on-time graduation (Allensworth & Easton, 2005; Allensworth, Morre, & Torre, 2014). While, student GPA in 6^th^ grade alone identified more than 30% of students at risk for not graduating high school (Allensworth et al., 2014). Parallel research with nationally representative samples finds that grade retention in K-8^th^ grade, in and of itself, is negatively associated with rates of high school graduation, and college enrollment and attendance (Andrew, 2014; Fine & Davis, 2003; Ou & Reynolds, 2010).

In addition to examining a student’s risk for grade retention, we also examine children’s likelihood of suspension in the 6^th^ grade. As students progress through school they must navigate increasingly complex social and academic settings, which many CPS students report feeling ill-equipped to manage (Roderick, Nagaoka, & Coca, 2008). Students that, have negative peer or teacher interactions, struggle in their coursework, and lack the skills to overcome such challenges may become emotionally reactive or avoid school altogether (see discussions in Balfanz, 2009; Balfanz & Brynes, 2012). These “behavior infractions”, even when relatively minor, can result in suspension, thus decreasing the likelihood a student will graduate high school on-time (Balfanz, 2009; Raffaele Mendez, 2009). In sum, we look at out of school suspension (hereafter referred to as OSS) in addition to risk for retention as student behavioral and academic problems many times co-occur and when they do, can exacerbate the negative consequences of each on longer term student outcomes (Balfanz, Herzog, & MacIver, 2007).

### 1.2 Cumulative Risk and the Multi-Dimensionality of Poverty

Drawing from research describing the environmental contexts children are exposed to over time, we know that families with low household income experience risks that spill across multiple dimensions. All 7 dimensions of poverty-related risk examined in the current study have been shown to negatively impact human development. Specifically, risk indicators included in the current study pull from family health risk, household economic risk, financial strain, interpersonal conflict, household composition, and housing/neighborhood risk. Parents with low household income often report decreased physical and mental health compared to their higher income peers, as well as higher relationship and family conflict, both of which are damaging to child development and learning (e.g., Clark-Nicolas & Gray-Little, 1991; Conger et al., 1990; Kotchick & Forehand, 2002; Lynch, Kaplan, & Shema, 1997). In addition, low-income families also report higher incidences of relationship instability, household member instability, and residential mobility, all of which negatively impact child development (Fomby & Cherlin, 2007; McCoy & Raver, 2014; Roy, McCoy, & Raver, 2014). To boost household resources, many low-income families “double up” with other adults (Kochhar & Cohn, 2011; Seltzer, Lau, & Bianchi, 2012; Weimers, 2014) resulting in crowded and chaotic households that come with their own costs for children (see, Sandstorm & Huerta, 2013, for review).

The cumulative risk literature has shown that as poverty-related risks accumulate in the lives of low-income families, so too does their negative impact on children (e.g., Gutman, Sameroff, & Eccles, 2002; Stoddard, Zimmerman, & Bauermeister, 2012). Exposure to high levels of cumulative risk in early childhood is associated with poorer educational outcomes, including decreases in student GPA and attendance through 12^th^ grade and greater internalizing and externalizing behavior problems in adolescence (Appleyard, Egeland, van Dulmen, & Sroufe, 2005; Gutman, Sameroff, & Cole, 2003). We draw from the cumulative risk framework (i.e., Rutter, 1979; Sameroff, Bartko, Baldwin, Baldwin, & Seifer, 1998) by including parental endorsement of 20 binary-risk indictors, spanning 7 dimensions of poverty-related risk in our measure of cumulative risk. Further, we averaged wave indices of cumulative risk as levels of risk are often stable over time whereby very few families report increased or decreased levels of risk (e.g., Sameroff et al., 1998). We expect that students exposed to higher levels of cumulative risk over time will have higher risk for retention and suspension in 6^th^ grade, as potential initial school readiness will dissolve in the face of continued and considerable adversity. It is to these measures of child academic and behavioral school readiness that that we now turn.

### 1.3 Implications of School Readiness

Students do not begin kindergarten on “equal footing” (e.g., Brooks-Gunn & Markman, 2005; Larson, Russ, Nelson, Olson, & Halfon, 2015). Rather, low-income children often lag behind their higher-income peers in math and reading, while simultaneously display more behavioral difficulties their higher income peers. Duncan and Magnuson’s (2011) study found lower income children were 1.34 and 0.63 standard deviations behind their higher income peers across measures of math and attention/engagement in kindergarten (respectively), and by 5^th^ grade these gaps increased to 1.38 and .68 respectively. In addition, the gap between lower and higher income student anti-social behaviors nearly doubled from 0.26 in kindergarten to 0.47 in 5^th^ grade. These findings suggest that, at best, low income students may remain just as far behind their higher income peers over time and, at worst, some low-income students may fall even further behind as they progress through school.

We examine early measures of student academic skills as well as their behavior problems prior to kindergarten entry as key predictors of their later risk for retention and OSS in the 6^th^ grade, given that a robust literature documents remarkable stability in these domains across development. For example, Duncan and colleagues (2007) found early math and reading skills were associated with later math and reading achievement through early adolescence (both of which are included within student risk for retention). Behavior problems at school entry are negatively associated with high school completion and college attendance (Duncan & Magnuson, 2011). Academic and behavioral problems also co-occur within children and when they do, jointly predict child performance across domains. In this case, children with both early behavioral and academic problems are more likely to have failing course grades and conduct problems in the 6^th^ grade compared to their peers with difficulties in just one, or neither, domain (Reinke et al., 2008). Thus, the current study builds on existing literature by examining associations between indicators of earlier (i.e., preschool) and later (i.e., 6^th^ grade) academic and behavioral outcomes, while also accounting for the different household contexts children are exposed to over-time.

### 1.4 Current Study

The current study examines whether exposure to poverty-related risk, above and beyond child school readiness prior to kindergarten, predicts child risk for retention and OSS in 6^th^ grade. We focus on risk for retention (i.e., whether they meet CPS promotion benchmarks), rather than observed cases of retention, as students who struggle across these domains will likely face hurdles on their path to graduation, regardless of whether or not they are actually retained (Allensworth et al., 2014; Nagaoka & Roderick, 2004). In addition we examine student OSS in 6^th^ grade as OSS may disrupt a student’s academic progress by, for example, decreasing feelings of connectedness to school or increasing the likelihood a student will drop out prior to high school graduation (McNeely, Nonnemaker, & Blum, 2002; Suh & Suh, 2007).

## 2. Method

### 2.1 Data and Sample

Data come from the Chicago School Readiness Project, a cluster-randomized control trial and longitudinal follow-up of 602 low-income children and their families, who attended 18 preschool programs within high poverty neighborhoods in Chicago. Parents provided information related to the number and types of poverty-related risks their families were exposed to during their child’s preschool (baseline), kindergarten (baseline + 1 year), third grade (baseline + 4 years), and fifth grade (baseline + 6 years), school-years. In addition we pull from CPS administrative data to code students’ risk for retention and observed suspension when child participants were in the 6th grade (baseline + 7 years).

Baseline characteristics of the sample are representative of the high poverty urban neighborhoods that surrounded the original Head Start sites. About 25% of parents reported having less than a high school level education when their child was in preschool, 70% reported living below the Federal Poverty Line, and 13% of children were reported as having low-birth weight (birth weight < 2500 grams). In addition, parents were on average 25-years old at the time of their child’s birth (*M* = 25.46, SD = 7.59), and the majority identified as being a racial/ethnic minority (i.e., African American, 70%; or Latino, 26%). Last, there were slightly more girl (53%) than boy child participants in the sample and children were on average about 4-years old at baseline (M = 49.16, SD = 7.38, months).

### 2.2 Analytic Sample

CSRP maintained high levels of participation across the four waves of data collection (ranging from 78-92% in follow-up waves). Of the full sample, 196 children (33%) were excluded from the analytic sample due to missing information related to their risk for retention or suspension. In addition, 49 cases (8%) missing more than 1 family survey wave were excluded from the analytic sample. Following these restrictions, our final analytic sample consists of 357 children and their families.

Mean difference tests (t-test for binary variables, and ANOVA for continuous variables) were examined between the analytic sample and the full sample regarding baseline child and family covariates, and child exposure to risks over time (full results available upon request). Results from these tests revealed fewer white/other race/ethnicity participants were included in the analytic sample compared to the full sample, yet no other significant differences between the samples emerged from the baseline characteristics. In later waves, more individuals in the analytic sample reported moving during the kindergarten wave of data collection, reported low savings in the 3^rd^ grade wave, and reported low partner support and trouble accessing medical care in the 5^th^ grade wave. The relatively few statistically significant differences between the samples suggest that the analytic sample is representative of the full sample.

### 2.3 Measures

#### 2.3.1 Outcome Measures

Student risk for retention is a binary variable that identifies whether students were at risk for being retained at the end of 6^th^ grade based on CPS promotion policies and existing research documenting the robust associations between on-track indicators and later student success (Allensworth et al., 2014). Specifically, students were given a value of “1” indicating they were at risk for retention if: their math or reading test scores on the Illinois Standards Achievement Test (ISAT) fell below the 24^th^ national percentile; they received a non-passing grade (i.e., a D or F) in an English/Language Arts or Math course or; the student had more than 9 unexcused absences. The majority of students within the analytic sample (65%) were at risk for being retained at the end of their 6^th^ grade school-year. We chose a risk for retention, rather than observed retention status, for two reasons. First, in practice, students can be promoted despite low grades, test scores, or attendance, and second, regardless of their retention status, a student’s inability to meet these basic academic or behavioral criteria is relevant for their chances of ultimately succeeding in school (Allensworth et al., 2014).

Student misconduct/suspension data were re-coded into one binary variable whereby a value of “1” indicates the student spent at least one day in OSS during the 6^th^ grade school-year. Over one-fifth (21%) of students in the analytic sample spent at least 1 day in OSS during the 6^th^ grade school-year.

### 2.4 Average Cumulative Poverty-Related Risk

At each wave parents reported whether their households experienced 20 binary risk indicators spanning 7 dimensions of poverty-related risk. Each risk was coded as a binary variable whereby a value of “1” indicates the presence of risk. Responses across the 20 binary risk indicators were summed to create four wave indices of cumulative risk, and from these four indices an average cumulative risk index was calculated by summing all wave indices and dividing by the total number of survey waves. Families missing more than 5 binary indicators within any wave (i.e., had fewer than 75% valid risk data each wave) were recorded to missing and a value was imputed using multiple imputation with chained equations. Families in the analytic sample reported just over 5 risks on average over time, and average cumulative risk scores ranged from about 0 to 11.5 risks over time. Specifically parents reported: Whether a family member has (1) an ongoing physical/mental health problem or (2) the parent has an ongoing physical/mental health problem (i.e., familial health risk); Whether parents are (3) unemployed, (4) have less than a high school diploma/GED, (5) have household incomes below the FPL, (6) have less than 1 month of savings, or (7) receive any government assistance (TANF, WIC, Food Stamps/SNAP, Medicaid /KidCare, housing assistance, free/reduced lunch, SSI, family support) (i.e., economic risk); Whether, (8) parents are single, (9) more than 3 children live in the household, or (10) more than 3 adults live in the household (i.e., household composition risk); Whether parents (11) cannot afford to do things for fun, (12) a family member has difficultly receiving medical care, or (13) they have difficulty paying bills (i.e., financial strain); Parents’ affirmative responses across three items (my partner or I “pushed, shoved, or slapped each other”, “showed respect [reverse coded]”, “insulted or swore at each other”) were aggregated and averaged, and individuals with scores in the highest quartile were identified as having high levels of partner conflict (14). Parents’ affirmative responses across two items (my partner or I “have gotten closer over time”, “suggested a compromise”) were aggregated and averaged and individuals with scores in the lowest quartile were identified as having low levels of partner support (15) (i.e., interpersonal conflict); Whether (16) a new child entered the household, (17) a new adult entered the household, or (18) the family moved in the past year (instability); and parents’ affirmative responses across six items gauging housing (e.g., “I do not have a working telephone at home”) or neighborhood problems (e.g., “there are abandoned houses in my neighborhood”) were aggregated and averaged and scores in the highest quartile indicated higher housing/neighborhood problems. (19) In addition parents reported whether, (20) a family member was assaulted or robbed (i.e., neighborhood/housing risk).

### 2.5 Child School Readiness

We also include characteristics of children just prior to kindergarten school entry (i.e., spring of the preschool year) to directly determine whether average cumulative risk is associated with child school success beyond indicators of their school readiness. We describe the measures of student behavioral and academic readiness briefly below (see, Li-Grining et al., in under review; Raver et al., 2011, for more detailed review).

Teachers reported child internalizing (α = .78) and externalizing (α = .90) behavior problems with the Behavior Problem Inventory (BPI; Peterson & Zill, 1986). Items within these subscales were aggregated and averaged (values range from 0 to 2) so that higher scores indicate more severe behavioral symptomatology.

Children’s early vocabulary skills were assessed with an adapted version of the Peabody Picture Vocabulary Test in English (PPVT-III; Dunn & Dunn, 1997) and in Spanish (TVIP, Dunn et al., 1986). Items were aggregated and averaged (values ranged from 0 to 1) whereby higher scores reflect greater vocabulary skills (α = .78). Children’s early math skills were directly assessed based on measures employed in the Early Childhood Longitudinal Study Kindergarten Cohort (ECLS-K; see, Zill, 2003). Again items were aggregated and averaged (values range from 0 to 1), whereby higher scores reflect greater early math skills (α = .82).

### 2.6 Covariates

Several parent report and time-invariant covariates were included in regression models in efforts to control for characteristics of children and their families linked to exposure to risk, child school readiness and later child academic outcomes. These covariates include, child gender (1 = boy), low birth weight (1 = < 2500 grams), treatment status (1 = received the CSRP intervention in Head Start), cohort (1 = 2004-2005 cohort; 0 = 2005-2006 cohort), age during preschool (years), whether the child’s mother was less than 20 years old at the child’s birth (i.e., 1 = teen mother), and parent reported race/ethnicity (white/other, Hispanic, African American [reference]).

### 2.7 Analytic Plan

The current study examines whether average cumulative risk is associated with student likelihood for retention and suspension in 6^th^ grade, net of child school readiness and other stable characteristics of children and families. We first calculate descriptive statistics related to children’s risk for retention and suspension in 6^th^ grade, other child and family characteristics and children’s academic and behavioral school readiness. In addition, we describe child exposure to 20 binary risk indicators which were aggregated and averaged to create a child’s exposure to average cumulative risk over time.

Next, using logistic regressions, we regressed child risk for retention on parent reported average cumulative risk to determine whether average cumulative risk is associated with a child’s risk for retention in 6^th^ grade. We then added stable characteristics of children and their parents to the model to examine whether cumulative risk is significantly associated with a child’s risk for retention above and beyond stable characteristics of children and their families. In the last step we included indicators of children’s academic (vocabulary and early math skills) and behavioral (internalizing and externalizing) school readiness in the spring of their preschool year, just prior to kindergarten, to determine whether characteristics of children or their environments matter more for a child’s risk of retention in 6^th^ grade.

We next examined whether or not average cumulative risk over time is associated with the likelihood a child will be suspended in 6^th^ grade. Consistent with the models described above, we regressed the likelihood for OSS in 6^th^ grade on average cumulative risk over time via a set of multivariate logistic regression models. We next added characteristics of children and their parents, and then indicators of child school readiness to models to determine whether characteristics describing children or their environments matter more when predicting school suspension. All statistical analyses were conducted using STATA version 12.1 statistical software and prior to conducting regression analyses, multiple imputation techniques with chained equations (ICE) were conducted to impute missing values on all variables in efforts to reduce bias in parameter estimates and standard errors (Graham, 2009; Graham & Schaefer, 1999).

## 3. Results

### 3.1 Descriptive Statistics

Table 1 presents sample descriptive statistics of children’s academic outcomes in 6^th^ grade and baseline covariates. Most children in the analytic sample were able to meet the math and reading achievement benchmarks necessary for promotion, with 91-92% receiving test scores above the benchmark in math and reading respectively. However, many children within the analytic sample struggled in their coursework: 33% received a non-passing grade in math and 44% received a non-passing grade in an English/Language Arts (ELA) course that year. Coming to school every day and staying in the classroom also appeared to present distinct challenges for our sample as, 14% of children had more than 9 unexcused absences and 21% spent at least 1 day in OSS. Taken together, 65% of the sample was at risk for retention at the end of their 6^th^ grade school-year, as CPS administrative policy required students to meet all three benchmarks to avoid summer school and being at risk for retention.

Moving to Table 2 we see rates of child exposure to 20 binary risk indicators, spanning 7 dimensions of poverty-related risk as reported by parents across the preschool, kindergarten, 3^rd^, and 5^th^ grade waves of the study. Overall, families in the analytic sample experienced considerable numbers of poverty-related risk over time and in the sentences that follow we examine a few of these trends in more detail. Eighty-seven percent of the sample reported receiving some form of government assistance during the preschool wave and this number increased to 95% of families in kindergarten, 96% of families in 3^rd^ grade, and 97% of families during the 5^th^ grade wave. This positive trend is in contrast to the number of families who reported household incomes below the FPL over time, as 80% of families lived below the FPL during the preschool wave and this number decreased steadily to 64% of the sample during the 5^th^ grade wave. Approximately half (54-60%) of CSRP families reported low savings at each wave, in all suggesting that increased income was not necessarily indicative of a complete reduction in financial strain. Regarding families’ neighborhood and housing risk, 8% of families reported that someone in the household was a victim of assault or robbery during the preschool wave and while this number fluctuated over time, it ultimately increased to 18% of families during the 5^th^ grade wave. Taken together, families in the analytic sample reported an average of 5 to 6 risks every wave of the study, with an overall average cumulative risk of just under 6 risks (*M =* 5.67, SD = 1.89) from preschool through 5^th^ grade.

### 3.2 Regression Results

Table 3 presents logistic regression results where we estimated whether average cumulative risk over time is associated with the likelihood a child is at risk for retention (Panel A, Model 1), the likelihood of receiving OSS (Panel B, Model 1), and net of baseline covariates (Model 2). In model 3, we also included indicators of child school readiness in the spring of the preschool year as key predictors of retention and out of school suspension (Model 3). Results from our baseline model (Model 1) indicate that average cumulative risk over time is positively and significantly associated with the likelihood a child will be at risk for retention in 6^th^ grade (OR = 1.26, p < .001). Increasing a child’s exposure to poverty-related risk over time by one risk on average is associated with a 26% increase in the likelihood a child will be at risk for retention in 6^th^ grade. This association remains statistically significant and decreases slightly (OR = 1.23, p < .01) when baseline characteristics of children and their families are included in the models (Model 2). Further, even when indicators of child school readiness (Model 3) are included in models, average cumulative risk from preschool through the 5^th^ grade remains positively and significantly associated with a child’s risk for retention in the 6^th^ grade (OR = 1.21, p < .05). In this case, net of child and family characteristics and indicators of school readiness, an increase in one risk on average over time is associated with a 21% increase in the odds children will be at risk for retention in 6^th^ grade. Looked at another way, one could expect 13% of children whose families reported no risks on average over time to be at risk for retention, compared to 40% of children whose families reported 6 risks on average over time (the mean level of risk in the analytic sample).

Beyond a student’s cumulative exposure to poverty-related risk over time, characteristics of children such as their school readiness, race/ethnicity, and age, also matter for their risk for retention in 6^th^ grade. Children with the highest vocabulary skills in preschool have 93% lower odds of being at risk for retention in 6^th^ grade compared to their peers with the lowest skills (OR = 0.07, p < .05). Holding all other variables constant, one could then expect 2% of students with vocabulary skills 1 standard deviation above the mean to be at risk for retention in 6^th^ grade compared to 5% of students with vocabulary skills 1 standard deviation below the mean in the spring of preschool. At the same time, children’s early math skills and behavioral school readiness are not significantly associated with the odds children are at risk for retention in 6^th^ grade.

Moving to Panel B of Table 3, we examine the association between average cumulative risk and the likelihood a child will be in OSS in 6^th^ grade and net of child and family covariates. Again, children’s exposure to higher average cumulative risk over the elementary school years is associated with greater odds they will be suspended in 6^th^ grade (Model 1). Increasing the average number of risks children are exposed to from preschool to 5^th^ grade by one risk is associated with a 22% increase in the odds the child will be suspended in 6^th^ grade (OR = 1.22, p < .01). Comparing this association to the same families as we did above this would mean 8% of students exposed to 0 risks on average are estimated to be suspended in 6^th^ grade, compared to 27% of students exposed to 6 risks on average over time (the average level of risk). This association is reduced, yet remains statistically significant, once characteristics of children and their families are included in models (Model 2). However, the association is reduced to non-significance once indicators of school readiness in the spring of preschool are included in the model (Model 3).

Instead, the academic and behavioral skills children demonstrated prior to their transition to kindergarten are statistically significant predictors of their later risk of OSS, but in ways that are mixed rather than consistent. Specifically, children with greater externalizing symptoms are 24% more likely to be suspended in 6^th^ grade (OR = 1.24, p < .001) compared to their lesser externalizing peers. Surprisingly, children’s manifestation of greater internalizing symptoms is a statistically significant and negative predictor of OSS so that students with greater internalizing behaviors in the spring of preschool are 37% less likely to be at risk for OSS than their lower internalizing peers (OR = 0.63, p < .01). In addition, children’s greater early math skills in the spring of preschool is significantly associated with decreased odds of suspension in 6^th^ grade, such that children with greater math skills are 85% less likely to be at risk for OSS than their lesser skilled peers (OR = 0.15, p < .10).

## 4. Discussion

Developmentally focused scholarship highlights that students from different socioeconomic strata do not enter kindergarten with the same academic and behavioral skills, and these gaps tend to increase as students move through elementary school (e.g., Duncan & Magnuson, 2011). A second body of literature finds that the out-of-school environments children are exposed to from birth through middle childhood can alternatively support or constrain children’s long term successes and potentially narrow or exacerbate these gaps (e.g., Duncan et al., 2011; Evans & Kim, 2012). Worse still, correlates of household poverty often accumulate in their negative impact on child and family well-being (Sameroff et al., 1998; Rutter, 1979). The current paper extends this research by explicitly integrating these two literatures to examine (1) whether children’s exposure to higher versus lower levels of cumulative risk from preschool through 5^th^ grade predict their risk for retention and OSS in 6^th^ grade and (2) whether cumulative risk predicts retention and OSS even after taking into account children’s early academic and behavioral skills as they make the transition to kindergarten.

### 4.1 Portrait of Academic Outcomes and Risks

Descriptive analyses of students’ risk of retention and suspension presented a mixed portrait of our sample’s successes and setbacks in 6^th^ grade. Students within the current sample were generally able to meet the standardized test score benchmarks for grade promotion. At the same time, many students struggled to meet their course grade benchmarks and a sizeable proportion of the sample demonstrated disengagement (i.e., 14% had > 9 unexcused absences) and misconduct (i.e., 21% received OSS) in the 6^th^ grade. These findings may not be entirely surprising when examined in light of the prevalence and stability of risks children in the current sample were exposed to over time.

No one dimension of poverty-related risk appears to drive the sample’s consistent levels of cumulative exposure to stressors. In any given wave, families’ cumulative indices of exposure to this wide range of stressors hovered, on average, between 5 and 6 risks over time. It is important to note that some families experienced only a small number of risks, and that the families in our sample experienced improvements in yearly income, on average, over that period. However it is important to note that while many families were able to move above the FPL, this success was not necessarily indicative of a net increase in financial stability as more families depended upon government assistance overtime and a little more than half reported low savings. Interpersonal conflict and exposure to violence were reported by a substantial fraction of the families in our sample over time, highlighting the toll that financial and economic insecurity can have on the emotional climate and safety of our sample. Nearly 20% of the sample reported exposure to violence in the 5^th^ grade wave and many parents consistently reported crowded households, strain and conflict in their relationships, and difficulties paying bills. This set of environmental measures highlights the utility of examining risk across multiple dimensions of poverty as diminished risk in one dimension was not necessarily mirrored across other dimensions.

Looking nationally, it is important to place the experiences of our current sample into a broader context. About halfway through the study, the nation entered one of the most severe economic downturns since the Great Depression. National rates of children living in poverty increased from about 17% in 2007 to about 23% in 2010 and have largely stabilized since (US Census Bureau, 2013). Increases in childhood poverty were likely a result of the nation’s unemployment rate doubling, a statistic that included many parents in the nation (Bureau of Labor Statistics, 2012, 2015; Isaac, 2013; Lovell & Isaac, 2010). Descriptions of poverty-related risk within our sample over this period thus echo the experiences of the nation as a whole as, for example, between 30-40% of parents in the sample reported unemployment over time while nationally, one third of parents lost their jobs during the recession (Isaac, 2013). Further, as the recession persisted through a prolonged recovery, we can see the initial gains many families made in some domains (i.e., household income) were undermined by persistent adversity in other areas. Increases, for example, in CSRP families’ experiences of neighborhood and housing risk (via increased violence exposure), and stable levels of interpersonal conflict, offer similar parallels to descriptions of risk that families experienced at the national level throughout the recession (see, Sandstorm & Huerta, 2013; Edin & Shaefer, 2015). It is no wonder then that children’s academic or behavioral success may become derailed as they are presented with numerous and continuous challenges to their safety and family well-being.

### 4.2 Struggles and Skills: Environments Versus School Readiness

We first examined whether parent-reported average cumulative risk from preschool through 5^th^ grade was associated with a child’s risk for retention and likelihood of OSS in 6^th^ grade. Our analyses clearly indicate that exposure to higher levels of poverty-related cumulative risk was associated with increased risk a child would be retained and suspended in 6^th^ grade. So that, child exposure to even just one additional risk, on average, from preschool through 5^th^ grade was associated with a 26% increase in the odds children were at risk for retention and 22% increase in the odds children were at risk for OSS in the 6^th^ grade. Further, higher levels of exposure to risk remained robust predictors of children’s retention and suspension in 6^th^ grade even after taking into account other child and parent characteristics at baseline. Specifically, even after taking these characteristics into account, each additional risk students were exposed to over time was associated with a 23% and 15% increase in the odds they would be at risk for retention or suspended in 6^th^ grade, respectively.

These results parallel prior scholarship exposing the impact, for better or for worse, the environments children are exposed to over time have on their thoughts, behaviors, and academic skills. The cumulative risk literature consistently finds child exposure to higher levels of cumulative risk, particularly in early childhood, can result in lower student academic performance and higher problem behaviors in middle childhood and adolescence (Gutman et al., 2003; Appelyard et al., 2005). Additionally, recent work with a diverse national sample finds that the early learning environment and parent’s educational beliefs explain 18 and 14% (respectively) of the gap between higher- and lower-income students’ early reading and math skills in kindergarten (Larson et al., 2015). In this regard we are not surprised that students in our sample exposed to higher numbers of cumulative risk over time were more likely to be at risk for retention or OSS in 6^th^ grade.

However, one bright note in this bleak story of risk is that the positive association between cumulative risk and risk for retention was attenuated, at least somewhat, after considering indicators of children’s own early academic and behavioral skills. Children who demonstrated greater vocabulary skills in preschool were less likely to be at risk for retention in 6th grade, even after taking into account their exposure to subsequent environmental risk. This finding extends literature documenting the benefits of early reading/literacy achievement in two ways (e.g., Cooper et al., 2014; La Paro & Pianta, 2000). First, beyond linking risk to measures of achievement alone (as in Cooper et al., 2014), we found early vocabulary skills are significantly associated with decreases in students’ later risk for retention, a construct that includes reading and math achievement as well as student attendance and course grades. And second, we found the association remained above and beyond the cumulative risky environments children were exposed to from preschool through 5th grade. We view these findings as compelling evidence for targeting low-income children’s early vocabulary skills as a potential mechanism to alter student’s longer term academic success.

When we included indicators of school readiness (including children’s early externalizing behavior problems) into models examining risk for OSS in 6^th^ grade, we find that the role of poverty-related risk is no longer as robust. Rather, children who demonstrated greater math skills in preschool and higher internalizing behavior problems were significantly less likely to receive OSS than their lesser-skilled or less internalizing counterparts, net of their exposure to poverty-related risk through elementary school. In contrast, students demonstrating higher externalizing behavior problems were more likely to be suspended in 6^th^ grade compared to their peers who demonstrated lower externalizing behaviors. These findings are surprising in some ways, and less so, in other ways.

First, how are these findings surprising? We are frankly somewhat surprised that the statistical power to detect the link between poverty-related risks and later OSS was attenuated once we included children’s early school readiness skills prior to kindergarten in our models. We were also surprised that children’s early math skills and early internalizing difficulties (as rated by teachers) would be predictors of lower likelihood of later risk of OSS in 6^th^ grade. Our findings align with correlational studies among nationally representative samples that highlight the benefits of supporting children’s early math skills for their later reading and math achievement (e.g., Duncan et al., 2007) and high school graduation (e.g., Duncan & Magnuson, 2011). To our knowledge this is the first study to find a significant association between student’s early math skills and a measure of their behavioral difficulty in formal school settings during middle school. To help inform future prevention and intervention efforts targeting the school conduct of low income students, more work should examine the potential benefits early math skills have for student behavior, perhaps via measures reflecting pro-social behavior or positive outcomes. Third, it was surprising to us to find that children who were reported by their teachers as having higher internalizing behavior problems in the spring of preschool were significantly less likely than their lower-internalizing peers to be suspended in 6^th^ grade. This association is easier to understand in light of the item content of this scale, whereby teachers reported whether children were “withdrawn”, or “crie[d] too much”. Although these behaviors are concerning, they are not necessarily the distracting or disruptive behaviors that could lead a student to OSS (Bosacki et al., 2011).

In other ways, it was not surprising to us to find that the students in our sample with higher externalizing behavior problems in spring of their preschool years continued to have behavioral difficulty in middle school, as reflected by their higher odds of being suspended for at least 1 day in the 6^th^ grade compared to their lower externalizing peers. This finding is mirrored in discussions throughout much developmental literature describing the stability, for better or worse, of some domains over development (e.g., Duncan & Magnuson, 2011; Reinke et al., 2008). That is, young children who are rated by teachers or parents as having higher externalizing behaviors continue to display these behaviors throughout school. Similarly, prior research suggests that students who are suspended from school tend to display similar disruptive behaviors earlier in their development (Balfanz, 2009). This suggests that perhaps, without intervention, behavior problems, much like environmental risk, accumulate negatively as students’ progress through school.

### 4.3 Limitations and Future Directions

Findings from the current study should be understood within the context of its limitations. First, the current study did not examine what processes may explain these relationships. Rather, we first used a set of empirical lenses focusing primarily on description and prediction to consider the powerful influence of children’s exposure to a host of poverty related “toxic stressors” as well as their early resources when predicting their later educational outcomes. A future direction of our research will be to examine what family or school processes may explain the links between both family poverty-related risk and child academic outcomes or early child skills and later academic outcomes. The current study is a first step to determining, explicitly, the roles that students’ environments and skills have for their later academic success and setbacks.

Second, the current study operationalized environmental risk via averaging the parent-reported cumulative risk indices at four different time points from the preschool through 5^th^ grade school-years. Emerging research documents the differential relationships between types or clusters of risk and student outcomes (Roy & Raver, 2014) as well as the importance of considering the role of the stability or instability of risks over time (i.e., Roy et al., 2014; Wolf et al., 2014). In future work we would like to examine variations in the types of risks families experienced over time, within the 7 dimensions of poverty operationalized within the paper. And further, we would like to examine whether exposure to different patterns of these risks over time are related to differential levels of child and family well-being.

Last, although the current study employs multiple respondents of children’s skills and environments, as well as a rich set of covariates that are correlated both with the presence of risk and the likelihood of grade repetition and OSS, there may still be unmeasured factors that explain these associations. Children and families are not randomly assigned to the environments they live in, and characteristics that are associated with the presence of environmental risk also likely explain child academic skills at school entry, as well as their behavior and skills later in development. While only a random experiment could convey causal claims related to what may matter more for children’s later success (their school readiness skills or their environments), such a study would be difficult, if not impossible, for practical and ethical reasons. Still, we are confident in our study’s findings as correlational studies (e.g., Duncan & Magnuson, 2011) and large RCT interventions (e.g., Barnett et al., 2013) point to the value of supporting children’s acquisition of early cognitive and behavior skills as a way to boost their later academic success and undermine their exposure to poverty-related risk.

### 4.4 Conclusion

We place the findings from this study within the context of two broad literatures. First, our study parallels an expanding literature documenting the many “toxic stressors” that children are exposed to from school entry through their high school graduation (see Blair & Raver, 2012). Families in the current study consistently reported experiencing about 6 risks on average, across a 7 year period. These poverty-related risks matter greatly for children by placing them at risk academically and behaviorally during middle childhood. Findings from this study also highlight the potential early educational settings may have as a mechanism to boost the school readiness of low-income children, ultimately helping students overcome exposure to environmental risk. Drawing from the now classic demonstration intervention efforts (i.e., the Perry Preschool and Abecedarian Projects, Chicago Child Parent Centers) and more recent “at-scale” interventions (in Tulsa, OK, Boston, MA and New Jersey), there is a strong evidence base to suggest that student school readiness can be positively altered via high quality early childcare educational settings (e.g., Barnett et al., 2013; Belfield et al., 2006; Gormley et al., 2005). That is, high quality early childhood education programs can be a cost effective strategy for improving children’s school readiness and reducing their risk for later grade retention or suspension. Our study supports this work by highlighting the associations early academic and behavioral indicators of school readiness have with student risk for retention and suspension net of robust measures of environmental risk.

## Figures and Tables

**Table 1:** Sample descriptive statistics of academic outcomes and covariates (n = 357)

CPS promotion/retention benchmarks a	
Math ISAT- score below 24^th^ % tile (%)	9%
Reading ISAT- score below 24^th^ % tile (%)	8%
Did not pass math course (%)	33%
Did not pass English/ Language Arts course (%)	44%
Had more than 9 unexcused absences (%)	14%
At risk for retention (%)	65%
Received at least 1 day of OSS (%)	21%
Indicators of school readiness (spring preschool)	
Vocabulary skills *M* (SD)	0.42 (0.38)
Early math skills *M* (SD)	0.55 (0.18)
Internalizing Behavior Problems *M* (SD)	0.15 (0.20)
Externalizing Behavior Problems *M* (SD)	0.22 (0.25)
Controls	
Treatment status (% treated)	50%
Cohort (% in Cohort 2)	44%
Low birth weight (%)	14%
Gender (% boy)	46%
Race/Ethnicity	
White or Other	5%
African American (reference)	69%
Hispanic	26%
Age in preschool (years) *M* (SD)	4.08 (0.60)
Teen mother (%)	23%

a As of CPS policy thru October 2013 students had to have ISAT math and reading >= 24^th^ percentile, grades C or higher in math and reading and no more than 9 unexcused absences (policy our sample faced during their 6^th^ grade school-year).

**Table 2:** Sample descriptive statistics of environmental risk and risk volatility (n = 357)

	Preschool	Kindergarten	3^rd^ Grade	5^th^ Grade
Health Risk (%)				
Maternal health issue	10%	6%	12%	14%
Family member health issue	21%	13%	22%	31%
Economic Risk (%)				
Unemployed	40%	30%	38%	41%
Low education (< H.S.)	25%	24%	22%	21%
Household income < = FPL	80%	70%	64%	64%
Received government assistance	87%	94%	96%	97%
Low savings (< 1 month)	58%	55%	54%	61%
Household Composition (%)				
Single (no res. partner)	59%	50%	53%	60%
4+ children in household	26%	26%	28%	28%
4+ adults in household	3%	3%	5%	6%
Financial Strain (%)				
Cannot do things for fun	17%	19%	19%	21%
Difficulty with medical care	11%	9%	14%	19%
Difficulty paying bills	39%	33%	51%	58%
Interpersonal Conflict (%)				
High relationship conflict	24%	25%	29%	20%
Low partner support	25%	30%	29%	34%
Household Instability (%)				
New child in household	14%	18%	12%	15%
New adult in household	7%	3%	9%	11%
Moved in past year	25%	20%	27%	27%
Housing/Neighborhood Risk (%)				
Housing/NB issues	23%	24%	32%	29%
Someone assaulted/ robbed	8%	5%	10%	18%
	Aggregate measures of risk			
Wave cumulative risk *M* (SD)	5.75 (2.15)	5.07 (2.18)	5.90 (2.60)	6.40 (2.63)
Average cumulative risk *M* (SD)		5.67 (1.89)		

**Table 3:** Predicting risk for retention and OSS by risk and school readiness (n = 357)

	Model 1		Model 2		Model 3	
	OR (SE)	[95% CI]	OR (SE)	[95% CI]	OR (SE)	[95% CI]
*Panel A: Student Risk for Retention*						

Average risk	1.26 (0.08) ***	[1.11, 1.43]	1.23 (0.08) **	[1.07, 1.40]	1.20 (0.09) *	[1.04, 1.41]
School Readiness						
Vocabulary	---	---	---	---	0.07 (0.08) *	[0.01, 0.58]
Math	---	---	---	---	0.27 (0.24)	[0.05, 1.57]
Internalizing	---	---	---	---	0.93 (0.09)	[0.78, 1.11]
Externalizing	---	---	---	---	1.07 (0.05)	[0.98, 1.17]
Treatment (Tx)	---	---	0.89 (0.21)	[0.55, 1.43]	0.86 (0.23)	[0.51, 1.45]
Cohort (1)	---	---	0.87 (0.26)	[0.48, 1.56]	0.92 (0.31)	[0.48, 1.77]
Low birth wght	---	---	1.42 (0.56)	[0.66, 3.07]	1.21 (0.49)	[0.54, 2.69]
Gender (girl)	---	---	0.65 (0.16)+	[0.40, 1.06]	0.76 (0.20)	[0.45, 1.28]
Race/ethnicity						
Black (ref.)	---	---	---	---	---	---
White/Other	---	---	0.33 (0.18) *	[0.11, 0.98]	0.28 (0.17) *	[0.08, 0.94]
Hispanic	---	---	0.49 (0.16) *	[0.26, 0.92]	0.36 (0.13) **	[0.18, 0.73]
Preschool age	---	---	1.21 (0.26)	[0.79, 1.84]	2.71 (0.85) **	[1.46, 5.04]
Teen mother	---	---	1.04 (0.32)	[0.57, 1.90]	0.94 (0.32)	[0.48, 1.84]
Constant	0.44 (0.16) *	[0.21, 0.92]	0.42 (0.43)	[0.06, 3.12]	0.13 (0.15)	[0.01, 1.28]

*Panel B: Likelihood of OSS*						

Average risk	1.22 (0.08) **	[1.06, 1.39]	1.15 (0.08) *	[1.00, 1.33]	1.12 (0.09)	[0.96, 1.32]
School Readiness						
Vocabulary	---	---	---	---	0.49 (0.62)	[0.04, 5.71]
Math		---	---	---	0.15 (0.17)+	[0.02, 1.39]
Internalizing	---	---	---	---	0.63 (0.09) **	[0.48, 0.83]
Externalizing	---	---	---	---	1.24 (0.06) ***	[1.13, 1.36]
Treatment (Tx)	---	---	1.45 (0.40)	[0.84, 2.50]	1.65 (0.53)	[0.37, 3.11]
Cohort (1)	---	---	0.87 (0.26)	[0.48, 1.58]	0.88 (0.31)	[0.40, 1.49]
Low birth wght	---	---	1.03 (0.39)	[0.48, 2.18]	0.95 (0.39)	[0.45, 1.74]
Gender (girl)	---	---	0.59 (0.17)+	[0.34, 1.03]	0.91 (0.29)	[0.48, 1.71]
Race/ethnicity		---				
Black (ref.)	---	---	---	---	---	---
White/Other	---	---	0.32 (0.25)	[0.07, 1.50]	0.27 (0.23)	[0.05, 1.41]
Hispanic	---	---	0.19 (0.10) **	[0.07, 0.54]	0.16 (0.09) **	[0.05, 0.50]
Preschool age	---	---	1.37 (0.32)	[0.86, 2.18]	2.36 (0.79) **	[1.23, 4.54]
Teen mother	---	---	0.95 (0.31)	[0.50, 1.80]	0.57 (0.22)	[0.27, 1.23]
Constant	0.08 (0.04) ***	[0.03, 0.19]	0.05 (0.05) **	[0.01, 0.42]	0.01 (0.02) **	[0.00, 0.18]

+ *Note*. p < .10,

* p < .05,

** p < .01,

*** p < .001,

OR = odds ratio, SE = standard errors, CI = confidence interval.
